# Psychology at the heart of every diabetes care intervention

**DOI:** 10.20945/2359-3997000000575

**Published:** 2022-11-28

**Authors:** Priscila Firmino Gonçalves Pecoli, Sergio Atala Dib

**Affiliations:** 1 Universidade Federal de São Paulo Escola Paulista de Medicina Departamento de Medicina São Paulo SP Brasil Centro de Diabetes e Endocrinologia, Divisão de Endocrinologia e Metabolismo, Departamento de Medicina, Escola Paulista de Medicina, Universidade Federal de São Paulo, São Paulo, SP, Brasil

Speculations about the individual aspects and their interfaces with the etiology and management of diabetes mellitus have generated a great amount of uncertainty and research across time. Investigators who focused their attention on the behavior of people with diabetes were intrigued by the significant differences in terms of their personal position about diagnosis and treatment when compared to the pattern of subjects with other conditions.

In 1679, Thomas Willis hypothesized about the importance of “prolonged sorrow” in the etiology of diabetes. Menninger, almost 250 years later, considered that depression and anxiety were characteristics of the “diabetic personality” pattern (1). In 1981, Dunn and Turtle broke down “the myth of a diabetic personality” by pointing out sampling bias on research which invalidated the generalization of specific findings from studies with this population. However, they concluded that “the clinical heterogeneity of diabetes is matched by the psychological heterogeneity of its sufferers” (2).

There is no doubt that the establishment of relationships between psychological, clinical, and physiological heterogeneities since the diagnosis of diabetes and across patients' life spans are complex and a never-ending field of research. Time after time, the investigation of psychological aspects of people with diabetes and family members, the interactions established with caregivers, personal barriers, and facilitators, including their own values, personality aspects, and coping strategies, have shown to be crucial to promote assertive interventions (3). This allows us to affirm that Psychology is in the heart of each intervention used in diabetes care.

In this issue of *Archives of Endocrinology and Metabolism (AE&M),* Gavazza and cols. (4), acknowledged Psychology as a central element, not merely as part of the supporting treatment, and presented an investigation of the associations between personality factors (PF) and health-related quality of life (HRQoL) by evaluating the treatment regimen (multiple daily injection [MDI] or insulin pump), the presence of type 1 diabetes (T1D) complications and the predominant PF.

By collecting data from a Public Health Assistance (PHA) research institution and a private clinic (PC), both located in Salvador, Bahia, Brazil, the authors concluded that the personalities of people with T1D influence their treatment and “neuroticism” was related to a better HRQoL. Their findings also brought us a great opportunity to reflect on healthcare settings assistance disparities and the relationship between gender differences and HRQoL.

Developing countries such as Brazil present a complex socioeconomic environment. Besides worrisome data correlated to financial spendings and to the number of subjects diagnosed with this chronic condition (5), barriers in accessing cutting-edge technologies and the best treatment options makes diabetes management even more challenging.

To overcome such struggles, patient-centered care approaches in similar socioeconomic contexts are more than welcome. They can empower people with diabetes in the coping process and promote the acceptance of self-management as an ally to the accomplishment of personal goals and the enjoyment of healthy lives (6).

Even being aware of the long journey that we have ahead of us, we can still make changes in the way we provide care by tracking and addressing psychosocial issues, as recommended in psychosocial care position statements (7–9) and advocating the presence of multidisciplinary teams in diabetes care institutions.

## References

[1] Menninger WC (1935). Psychological factors in the etiology of diabetes. J Nerv Ment Dis.

[2] Dunn SM, Turtle JR (1981). The myth of the diabetic personality. Diabetes Care.

[3] Due-Christensen M, Willaing I, Ismail K, Forbes A (2019). Learning about Type 1 diabetes and learning to live with it when diagnosed in adulthood: two distinct but inter-related psychological processes of adaptation A qualitative longitudinal study. Diabet Med.

[4] de Barreiros Gavazza MLN, Martins E, Netto, Ramalho ACR (2022). Association between personality factors and health-related quality of life in type 1 diabetes patients. Arch Endocrinol Metab.

[5] IDF Diabetes Atlas (2021). IDF Diabetes Atlas.

[6] Pecoli PFG (2021). São Paulo, 2021. 76 f. Dissertação (Mestrado em Endocrinologia e Metabologia) – Escola Paulista de Medicina.

[7] Rodrigues G, Malerbi F, Pecoli P, Forti A, Bertoluci M (2022). Aspectos psicossociais do diabetes tipos 1 e 2. Diretriz Oficial da Sociedade Brasileira de Diabetes.

[8] Young-Hyman D, de Groot M, Hill-Briggs F, Gonzalez JS, Hood K, Peyrot M (2016). Psychosocial Care for People With Diabetes: A Position Statement of the American Diabetes Association. Diabetes Care.

[9] Aroda VR, Bakris G, Benson G, American Diabetes Association Professional Practice Committee; American Diabetes Association Professional Practice Committee (2022). 5. Facilitating Behavior Change and Well-being to Improve Health Outcomes: Standards of Medical Care in Diabetes-2022. Diabetes Care.

